# Which irrigating provides a better bond strength in glass fiber posts: Chlorhexidine or Sodium Hypochlorite? A systematic review with meta-analysis

**DOI:** 10.4317/jced.60553

**Published:** 2023-08-01

**Authors:** Eneas-Pereira-da Silva Júnior, Sarah-Freitas Araújo, Cláudio-Paulo-Pereira de Assis, André-Rodrigo-Justino da Silva, Maria-Catarina-Almeida Lago, Rodivan Braz

**Affiliations:** 1 University of Pernambuco. Recife, state of Pernambuco, Brazil

## Abstract

**Background:**

The aim of this systematic review and meta-analysis was to evaluate the influence of the use of chlorhexidine on endodontic instrumentation on the bond strength of glass fiber posts with resin cements. The guiding question of the study was: “Is the bond strength of glass fiber posts greater when using chlorhexidine as an irrigator in endodontic treatment instead of sodium hypochlorite?”.

**Material and Methods:**

This study was conducted according to Guidelines of the Preferred Reporting Items for Systematic Reviews and Meta-analysis (PRISMA). In vitro studies were included that have compared chlorhexidine and sodium hypochlorite as an endodontic irrigator in the chemical-mechanical preparation.

**Results:**

Eight studies were included in the qualitative analysis and seven in the meta-analysis. Both the analysis by subgroups and the total analysis, using a random effect model, showed no statistically significant differences between the groups (*p*> 0.05), either in the specific analysis for cervical root third (*p* = 0.30; 95% CI = -2.11, 6.91) or medium (*p* = 0.05; 95% CI = -0.03, 4.56), or nonspecific regarding the third to the root third (*p* = 0.48; 95% CI = -4.00, 1.86).

**Conclusions:**

There are no statistically significant differences in the bond strength of glass fiber posts and resin cements in teeth endodontically treated under irrigation by both chlorhexidine and sodium hypochlorite.

** Key words:**Chlorhexidine, sodium hypochlorite, bond strength, glass fiber post.

## Introduction

Root canal treatment is usually associated with extensive coronary destruction resulting from caries or cavity preparation. In these situations, in which there is a decrease in the modulus of elasticity and structural and biochemical changes in dentin, or when the remainder is no longer sufficient to retain and stabilize the restoration, there is a greater risk for the good prognosis of the restorative treatment, and a post may be indicated to stabilize the coronary portion (1).

Glass fiber posts, beyond having better mechanical properties (2), can be associated with adhesive systems. However, adhesion to the root dentin substrate is more challenging due to the high “factor C”, which can contribute to an inappropriate polymerization contraction (3). The root dentin also has several structural differences in relation to the coronary, such as the fact that it is more sclerotic, with fewer tubules and eventually obliterated, especially after the 3rd decade of life (4,5).

Additionally, several factors can interfere with adhesion, whether with conventional resin cements associated with adhesive systems, or self-adhesive. One of them is the irrigating solution used in the chemical-mechanical preparation of the root canal. Sodium hypochlorite has been chosen by most endodontists as the main auxiliary chemical substance in endodontic therapy. However, one of its mechanisms of action is protein denaturation, which in the case of dentin can be a critical factor, since it modifies the collagen fibrils that are essential for the formation of the hybrid layer. Furthermore, because it is an oxidizing solution, the residual oxygen generated by the dissociation of its molecule can inhibit the polymerization of adhesive agents (6).

Chlorhexidine has been shown to be an irrigating solution with an antimicrobial potential similar to that of sodium hypochlorite (7). However, regarding the residual effect of dentin as an adhesive substrate, studies have pointed out chlorhexidine as an important inhibitor of metalloproteinases that are trapped in the dentin matrix and are responsible for the collagen degradation (8,9). However, this solution may even have a deleterious effect on the bond strength and the permeability between cement and dentin (10). This may be due to the reaction between chlorhexidine and dentin phosphate resulting in precipitation, which can form a physical barrier between adhesive and dentin. Because it is an alternative solution to sodium hypochlorite, it is important the endodontists know about these properties when selecting the chemical irrigator not only in preparing the space for the post, but from the chemical-mechanical preparation of the root canal of a dental element that will be destined rehabilitation with glass fiber post.

Thus, the aim of this systematic review was to evaluate the influence of the use of chlorhexidine on endodontic instrumentation on the bond strength of glass fiber posts with resin cements. The null hypothesis tested in this systematic review was that there are no significant differences between the bond strength values of glass fiber posts to the root dentin of conducts irrigated with chlorhexidine and sodium hypochlorite.

## Material and Methods

This systematic review was conducted according to Guidelines of the Preferred Reporting Items for Systematic Reviews and Meta-analysis (PRISMA) checklist (11) and was registered on the International Prospective Register of Systematic Reviews (PROSPERO) under registration number CRD442020203848.

The guiding question of the study was: “Is the bond strength of glass fiber posts greater when using chlorhexidine as an irrigator in endodontic treatment instead of sodium hypochlorite?” based on the PICO criteria. The population (P) consisted of teeth treated endodontically with an intraradicular retainer; intervention (I) was endodontic irrigation with chlorhexidine; Comparison (C) endodontic irrigation with sodium hypochlorite; and as a result (O) the bond strength of fiber posts cemented with resin cement.

The inclusion criteria used were laboratory studies that have compared the effect of the use of chlorhexidine and sodium hypochlorite during the chemical-mechanical preparation of the root canal on the strength of adhesion of fiberglass posts to dentine. The exclusion criteria were: studies that have not compared in the irrigant in the stage of chemical-mechanical preparation of endodontic therapy; studies that have not informed the irrigating solution used in this stage of the root canal treatment; clinical trials; literature reviews; laboratory tests that have not measured the bond strength of glass fiber posts.

The electronic search in the literature was done by two researchers, working independently. The studies were selected according to the inclusion / exclusion criteria, based on the title of the article and abstract in the following databases: PubMed / MEDLINE, Scopus, Web of Science and Cochrane Library, using the terms described in Table 1. The research was carried out until April 2021 without limiting the year of publication or language. Studies that were not clear whether they would meet the inclusion / exclusion criteria were downloaded and read in full, and then the decision was made to include them or not in the review. In case of divergence between the researchers, a third researcher analyzed all the differences in the choice between the researchers and a consensus was reached.

**Table 1:**
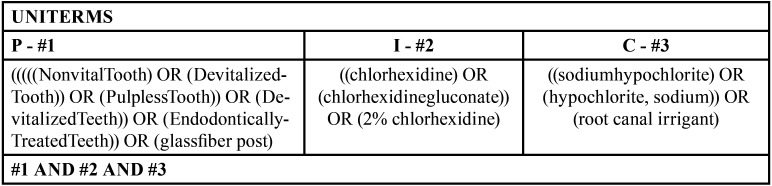
Uniterms.

Important information from the articles was collected and independently reviewed by the two authors. A careful analysis was conducted to check for disagreements between the authors, and a third author was consulted when there was no consensus.

**Figure 1:**
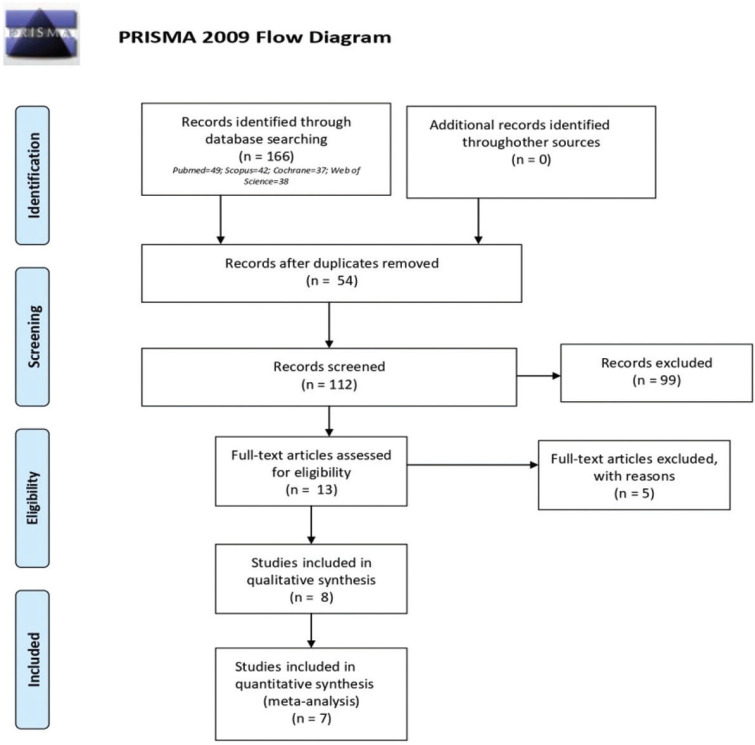
PRISMA. Flow Diagram.

Figure 1 shows the flowchart of the search, screening and selection of articles. The search strategy reached 166 articles, of which 54 were removed because they were in duplicate. The 112 studies were screened by title and abstract. At the end, 13 articles (10,12-23) were selected for the analysis of the full text. At this stage, 5 articles were excluded for the following reasons: 3 articles (10,12,13) because the irrigating variable was only applied in the preparation of the space for a post, and not in the chemical-mechanical preparation of endodontic treatment; one (14) for not having assessed the adhesion of post; and 1 (15) because it was not possible to access the text completely, either through the magazine or through contact with the authors. Finally, 8 articles were included (16-23) in the systematic review.

The methodological quality of the included studies was assessed as previously described by Astudillo-Rubio *et al*. (24), and according to the Checklist for Reporting In vitro Studies (25). It was verified whether the bond strength was tested following the parameters: 1. Standardization of the sample; 2. Single operator; 3. Description of the sample calculation; 4. Blinding the test machine operator; 5. Design of the bond strength test according to standards and specifications. If the article reported clearly about the parameter, it received a score of 0; if a specific parameter was reported, but insufficiently or unclearly, the score was 1, and if it was not possible to find this information, the score was 2. Articles with a total score between 0 and 3 were classified as low risk of bias; those with scores from 4 to 7 as moderate risk and scores from 8 to 10 as high risk.

The Kappa coefficient test was used to calculate the level of concordance between authors during these lection processes of the articles in the PubMed / MEDLINE, Scopus and The Cochrane Library databases. Any disagreements were resolved by discussion and the consensus of all authors.

The differences between the means of the bond strength measures of the groups were used as an effect measure for the quantitative analysis of the selected studies. The meta-analysis was based on the Mantel-Haenzel (MH) and inverse variance (IV) methods. Chlorhexidine and Hypochlorite were used in the study to assess the effect soft bond strength. The Confidence Interval (CI) of 95% were calculated for each study and were considered significant when *p* <0.05. The extracted data were analyzed using Review Manager Software (RevMan) 5.4.1 (The Cochrane Collaboration, Copenhagen, Denmark).

Due to the heterogeneity between the studies, it was possible to divide the meta-analysis into 3 subgroups: studies that presented data for tests on the cervical third separately; studies that presented data for tests in the middle third separately; and studies that presented their results in general, without division by root third.

## Results

The selected studies were all *in vitro* tests carried out between the years 2014 and 2020. Of these, 6 were performed in Brazil, 1 in Croatia and 1 in India (Table 2-2 cont.-2). Of the selected researches, 4 used bovine teeth (16,18-20), 4 used uniradicular human teeth (17,21-23). The samples remained stored in 0.1% thymol in 1 study (18); in distilled water in 3 (16,20,22) , and 2 in chloramine-T: Katalinic, Glover & Anic (17) choose a concentration of 5%, while Kaif & Bissu (23) for 0.5%). 1 work (19) did not report the storage method. Table 2- Table 2 cont.-2 contains the main conclusions of the studies evaluated. In addition to irrigating solutions, Santana *et al*. (16) evaluated the effect of manual chemical-mechanical preparation with steel files and mechanized nickel-titanium files, immediately after cementation, or after 2 months in storage for artificial aging. Two cross-sections of 1 mm from each third of each sample were submitted to the push-out test. Only in the cervical third of the teeth whose endodontic instrumentation was performed manually and stored for 2 months before the test did they observe lower values when irrigated with chlorhexidine. In the other circumstances, no significant differences were observed.

**Table 2:**
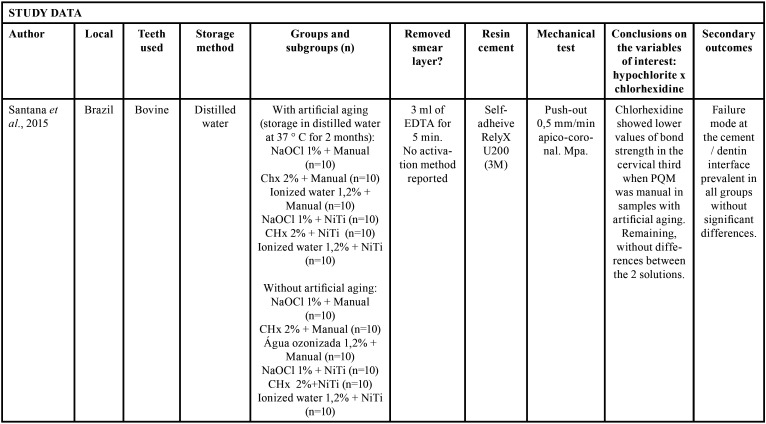
Study data.

**Table 2 cont.**
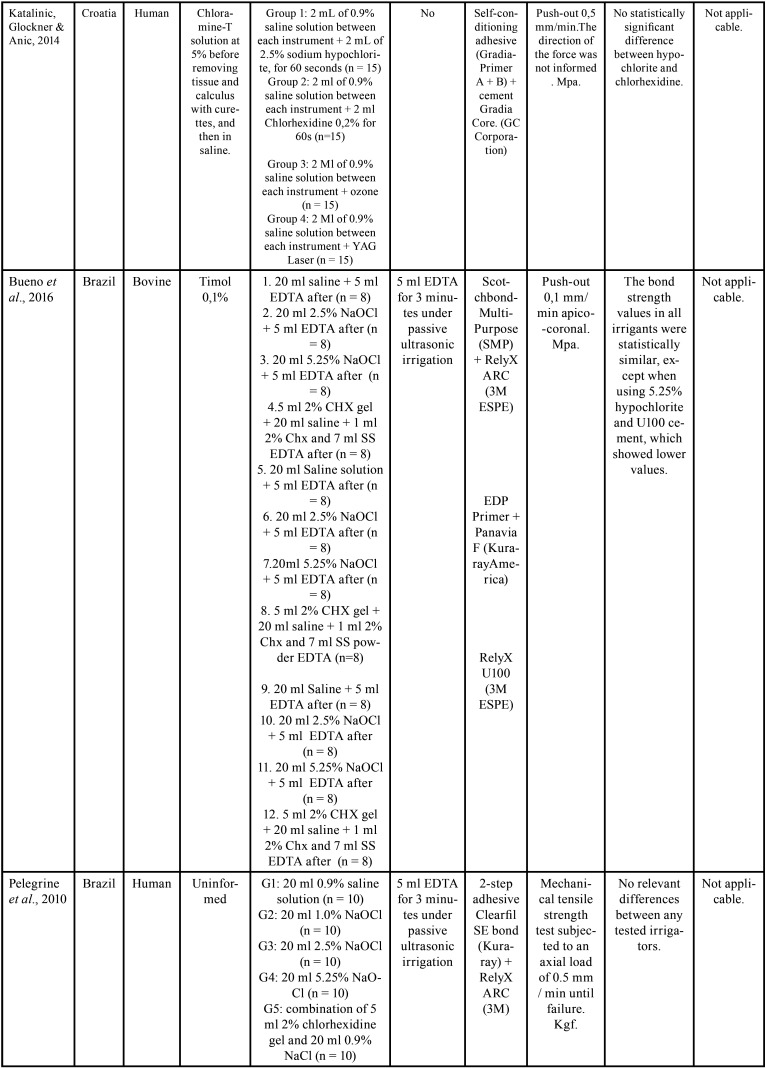
Study data.

**Table 2 cont.-1:**
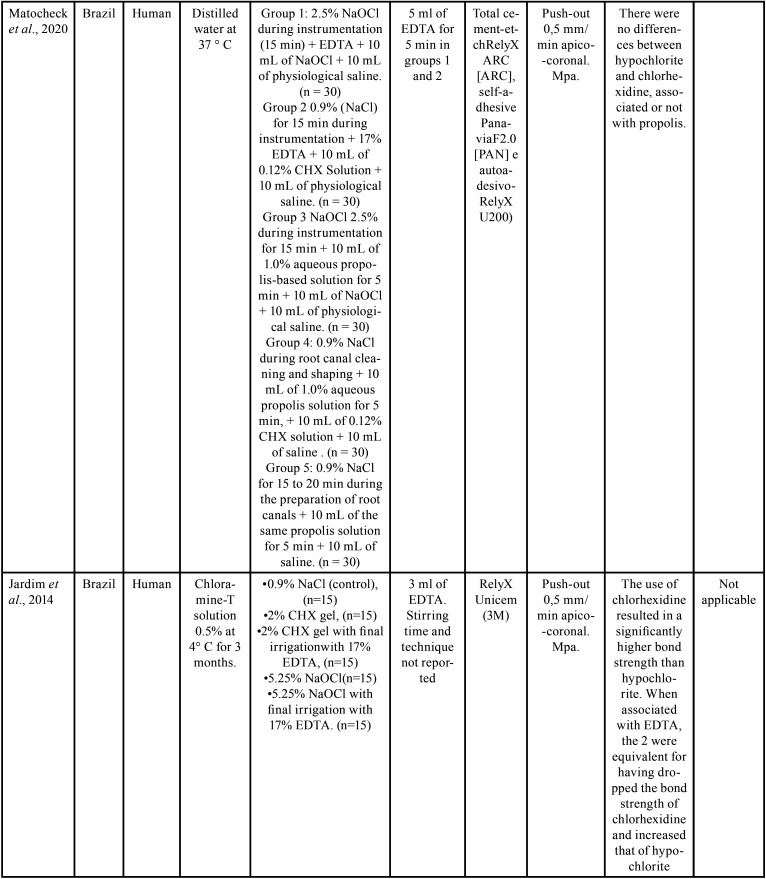
Study data.

**Table 2 cont.-2:**
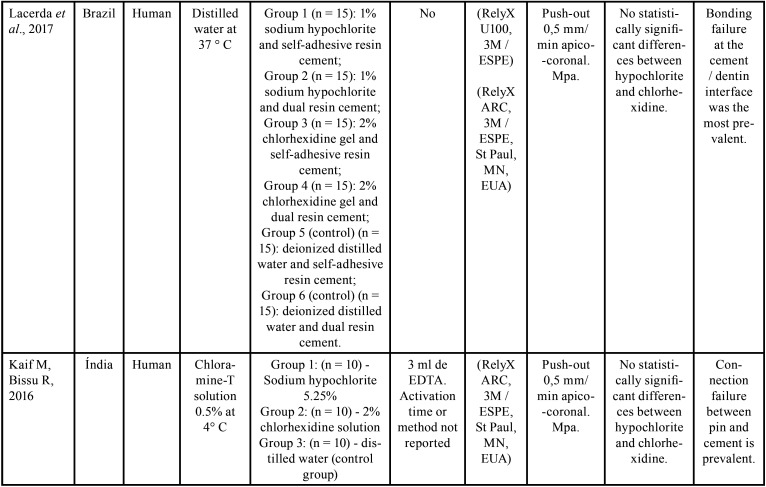
Study data.

Katalinic, Glocker & Anic, Pelegrine *et al*. (17), Matocheck *et al*. (20), Lacerda *et al*. (22), and Kaif & Bissu (23) did not observe differences in bond strength between samples irrigated with chlorhexidine and sodium hypochlorite under any circumstances.

Bueno *et al*. (18) compared chlorhexidine with 2.5% and 5.25% sodium hypochlorite. They observed significantly lower bond strength values only in samples whose endodontic irrigation was done with 5.25% hypochlorite and in which the posts were cemented with RelyX U100 self-adhesive cement.

Jardim *et al*. (21) also used sodium hypochlorite at a concentration of 5.25%, confirming its inferiority in relation to chlorhexidine only when smear layer was not removed, showing equivalence between the two when using 17% EDTA solution at the end of the chemical-mechanical preparation. Table 3,  Table 3 cont. describes the results of the bond strength tests of the selected studies.

**Table 3:**
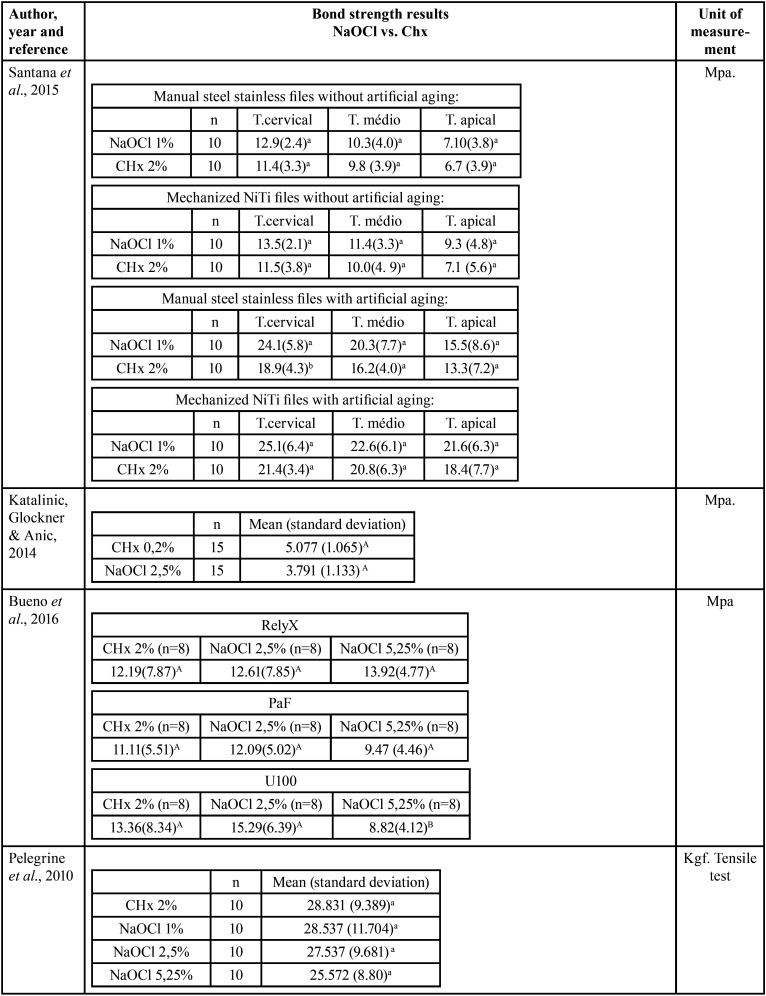
Results of the Bond strength tests with the variables of interest in this study.

**Table 3 cont.**
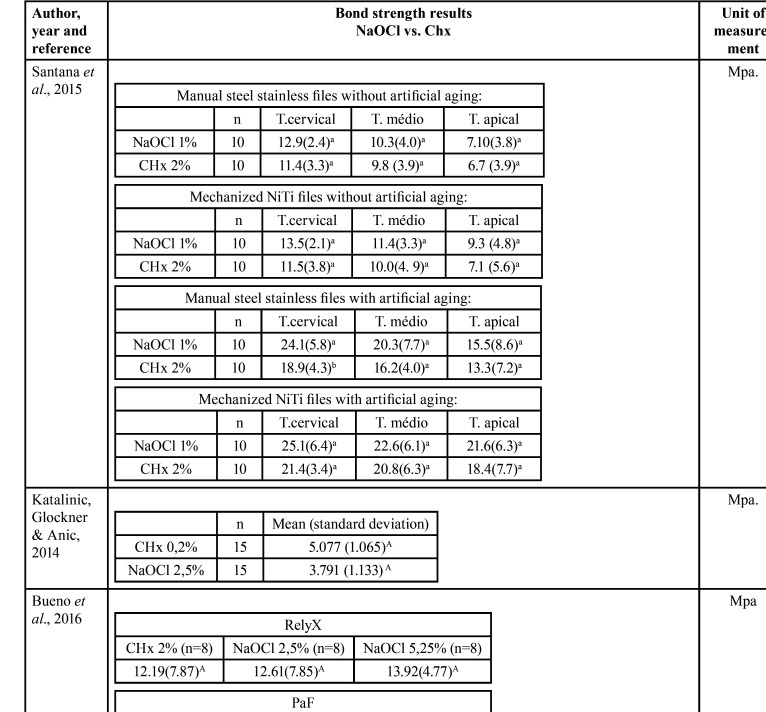
Results of the Bond strength tests with the variables of interest in this study.

Three studies evaluated using a microscope the type of failure that was most prevalent (Table 2,  Table 2 cont.-2). Santana *et al*. (16) and Lacerda *et al*. (22) observed that adhesive failure at the cement / dentin interface was the most prevalent, regardless of the type of endodontic solution used. Kaif & Bissu (23) found that most of the failures in their samples submitted to tests occurred at the interface between post and cement. Table 4 shows the classification of risk of bias in each study, with all studies showing moderate risk according to the classification adopted.

**Table 4:**
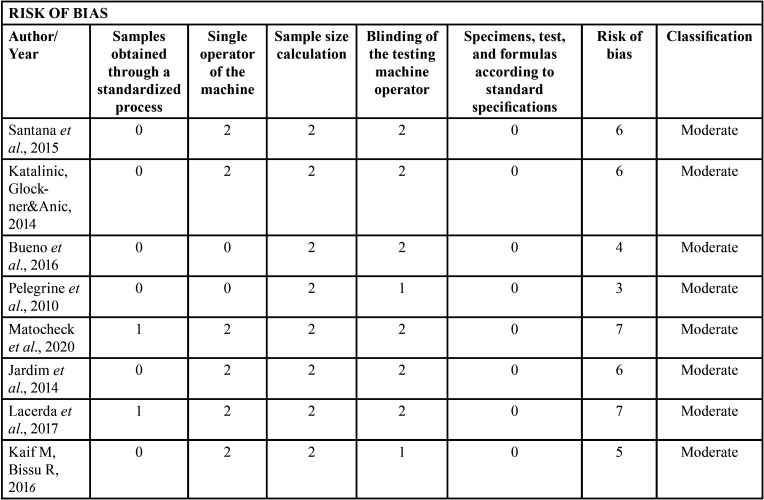
Analysis of the risks of bias.

**Figure 2:**
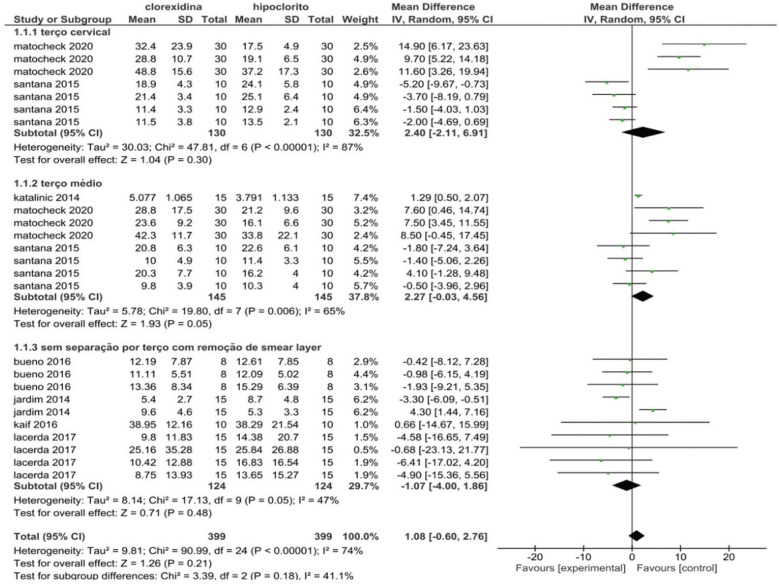
PRISMA.

Both the analysis by subgroups and the total analysis, using a random effect model, showed no statistically significant differences between the groups (*p*> 0.05) (Fig. 2), either in the specific analysis for cervical root third (*p* = 0.30; 95% CI = -2.11, 6.91) or medium (*p* = 0.05; 95% CI = -0.03, 4.56), or nonspecific for the third to the root third (*p* = 0.48; 95% CI = -4.00, 1.86).

## Discussion

The qualitative and quantitative analyzes of the studies included in this systematic review showed that there are no significant differences between the values of bond strength of fiberglass posts to the root dentin of channels irrigated with chlorhexidine and sodium hypochlorite, confirming the hypothesis tested, in addition to be in line with other studies (16-23). The study by Pelegrine *et al*. (19) was excluded from the meta-analysis because it used a tensile test to measure the bond strength of fiberglass posts. All others used a push-out test.

Endodontic treatment causes changes in the intraradicular dentin structure, and studies have shown that sodium hypochlorite can modify the structure of collagen, in addition to its residual oxidizing effect that can interfere with polymerization reactions (26). This issue becomes a concern due to the fact that hypochlorite, in concentrations ranging from 0.5 to 6%, is the most used solution in endodontic practice because it combines characteristics such as high antimicrobial power (27), in addition to being an excellent solvent for pulp organic matter and its low cost.

Chlorhexidine has shown antimicrobial efficacy similar to sodium hypochlorite (28) low cytotoxicity and high substantivity, which have made it the irrigating solution of choice for many endodontists. In addition, properties such as inhibition of metalloproteinases that can act in the collagen degradation (14,29), have made these authors, in an attempt to optimize the adhesion and the good prognosis of the rehabilitation of these teeth, indicate its use in dentin irrigation prior to adhesive procedures.

Regarding the bond strength of glass fiber posts, the results of this review indicate that the use of chlorhexidine or sodium hypochlorite as an endodontic irrigator generates significant differences only in specific situations, such as in the use of high concentrations of hypochlorite associated with self-adhesive cements. The studies are heterogeneous among themselves, because in addition to irrigating solutions, they apply other independent variables, such as chemical-mechanical preparation technique, use of EDTA, sample storage time, among others.

Most studies used the push-out test to measure the bond strength of post and cement to dentin because it allows the best use of the sample and its analysis separately by thirds of the root (30), which does not it is possible in mechanical tensile tests, in addition to more accurately predicting a clinical situation because the force is applied more homogeneously on the post and has lower rates of pre-mature failures of the samples (31).

According to the studies of Katalinic, Glocker, Anic (17), Pelegrine *et al*. (19), Matocheck *et al*. (20) and Lacerda *et al*. (22), the use of chlorhexidine in endodontic treatment does not imply additional bond strength in relation to sodium hypochlorite in cases where it is used in concentrations of 1% and 2.5%.

The work by Santana *et al*. (16) showed equivalence in the bond strength values using self-adhesive cement in several clinical simulations performed *in vitro*, except when using manual instrumentation under chlorhexidine irrigation, followed by artificial aging. In this case, they presented lower bond strength values, but only in the cervical third of the evaluated samples. The small taper of manual endodontic instruments makes it difficult to touch the dentinal walls in the radicular cervical third, which is generally wider, during endodontic preparation. This may have contributed to an accumulation of dentin debris, given the low capacity of chlorhexidine to dissolve pulp organic content, especially in aged samples. This may have prevented the formation of an adequate number of chemical bonds from the self-adhesive cement to dentin in this specific part of the sample, where the cement line is thicker.

Jardim *et al*. (21) observed that the adhesion of self-adhesive cements (RelyXUnicem, 3M) in dentin irrigated with chlorhexidine gel 2% was higher in relation to NaOCL 5.25%. However, when instituting the final irrigation protocol with 17% EDTA, the bond strength data became equivalent, as a reduction in values was observed in samples irrigated with chlorhexidine. Baldion *et al*. (32) stated that the use of chelating substances in the preparation of the post space can negatively interfere in adherence. Santos *et al*. (2006) found that the combination of chlorhexidine gel and EDTA 17% led to a drop in the bond strength values of a resin (Filtek Z20, 3M Espe) with universal adhesive (Clearfil SE Bond, Kuraray, Kurashiki, Japan) in comparison to the isolated use of chlorhexidine. This effect was attributed to the fact that, even though it is water-soluble, the complete removal of the chlorhexidine gel from the channel is more difficult. The permanence of residues can lead to an association with EDTA and generate residual products that affect the proper infiltration of universal adhesives in dentinal tubules, which may explain the findings by Jardim *et al*. (21). Only the works of Katalinic, Glockner, Anic (17) and Lacerda *et al*. (22) did not remove smear layer after chemical-mechanical preparation of the root canal. The others used a 17% EDTA solution for this purpose.

On the other hand, Bueno *et al*. (18) found that the use of high concentrations of sodium hypochlorite, such as 5.25%, promotes a deleterious effect on the adhesion to self-adhesive cement (RelyX U100, 3M) in relation to chlorhexidine, even after passive ultrasonic irrigation with EDTA at 17 %. The adhesion mechanism of RelyX U100 is explained by dentin hybridization by acid monomers and by the chemical interaction between hydroxyapatite and cement (33). The high concentration of 5.25% may have caused, significantly, the degradation of the collagen of the dentinal structure, thus compromising hybridization, in addition to its oxidizing characteristic having interfered in the polymerization reactions, effects that in lower concentrations, such as 1 % and 2.5%, did not manifest themselves in a decisive way to the point of interfering in adherence. Differences in mechanical tests can also explain the differences in the results of these two studies, since the work by Jardim *et al*. (21), performed a test at a lower speed, of 0.5 mm / min, whereas Bueno *et al*. (18) executed the push-out test at 1 mm / min.

When using conventional adhesive systems (EDP Primer + Panavia F, (Kuraray America and Scotch bond Multi Purpose (SMP) + RelyX ARC, 3M ESPE), even at a concentration of 5.25%, the same work by Bueno *et al*. (18) showed no interference with dentin adhesion, as well as Pelegrine *et al*. (19) (Clearfil SE bond, Kuraray America + RelyX ARC (3M), and Kaif & Bissu (23) (RelyX ARC + Adper Single Bond 2, 3M ESPE).

Studies have also shown that the highest prevalence of adhesive failures occurs between cement and dentin, showing that is in fact the most fragile interface of adhesion.

Due to the fact that this is a review of *in vitro* studies, the results should be carefully analyzed, interpreted and extrapolated to clinical situations. All included studies were classified as moderate risk of bias. Only 2 studies (18,19) reported that a single operator manipulated all samples, and it was not informed or it was not clear in any study whether the operator of the mechanical testing machine was blinded. In addition, no study reported whether a sample calculation was made or how the sample size was obtained, which may be controversial about the power of the sample to generate statistically significant results.

As this is a review of laboratory tests, this study recommends the execution of prospective clinical studies on the predictability of rehabilitation with post of teeth endodontically treated with sodium hypochlorite and chlorhexidine with respect to bond strength.

## Conclusions

There are no statistically significant differences in the bond strength of glass fiber posts and resin cements in teeth treated endodontically under irrigation by both chlorhexidine and sodium hypochlorite. Prospective clinical studies on the predictability of rehabilitation with tooth post endodontically treated with sodium hypochlorite and chlorhexidine with respect to bond strength are recommended.
